# Transcriptomics analyses reveal the effects of Pentagamaboronon-0-ol on PI3K/Akt and cell cycle of HER2+ breast cancer cells

**DOI:** 10.1016/j.jsps.2023.101847

**Published:** 2023-10-27

**Authors:** Adam Hermawan, Febri Wulandari, Rohmad Yudi Utomo, Ratna Asmah Susidarti, Mitsunori Kirihata, Edy Meiyanto

**Affiliations:** a Laboratory of Macromolecular Engineering, Department of Pharmaceutical Chemistry, Faculty of Pharmacy, Universitas Gadjah Mada Sekip Utara II, 55281, Yogyakarta, Indonesia; b Cancer Chemoprevention Research Center, Faculty of Pharmacy, Universitas Gadjah Mada Sekip Utara II, 55281, Yogyakarta, Indonesia; c Laboratory of Advanced Pharmaceutical Sciences. APSLC Building, Faculty of Pharmacy, Universitas Gadjah Mada Sekip Utara II, 55281, Yogyakarta, Indonesia; d Laboratory of Medicinal Chemistry, Department of Pharmaceutical Chemistry, Faculty of Pharmacy, Universitas Gadjah Mada Sekip Utara II, 55281, Yogyakarta, Indonesia; e Research Center for BNCT, Osaka Metropolitan University, 1-2, Gakuen-cho, Naka-ku, Sakai, Osaka 599-8570, Japan

**Keywords:** PGB-0-ol, HER2+ breast cancer, Transcriptomics, Anticancer, Drug development

## Abstract

**Introduction:**

Monoclonal antibodies and targeted therapies against HER2+ breast cancer has improved overall and disease-free survival in patients; however, encountering drug resistance causes recurrence, necessitating the development of newer HER2-targeted medications. A curcumin analog PGB-0-ol showed most cytotoxicity against HCC1954 HER2+ breast cancer cells than against other subtypes of breast cancer cells.

**Objective:**

Here, we employed next-generation sequencing technology to elucidate the molecular mechanism underlying the effect of PGB-0-ol on HCC1954 HER2+ breast cancer cells.

**Methods:**

The molecular mechanism underlying the action of PGB-0-ol on HCC1954 HER2+ breast cancer cells was determined using next-generation sequencing technologies. Additional bioinformatics studies were performed, including gene ontology (GO), Kyoto Encyclopedia of Genes and Genomes (KEGG) pathway, disease-gene, and drug-gene associations, network topology analysis (NTA), and gene set enrichment analysis (GSEA).

**Results:**

We detected 2,263 differentially expressed genes (DEGs) (1,459 upregulated and 804 downregulated) in the PGB-0-ol- and DMSO-treated HCC1954 cells. KEGG enrichment data revealed the control of phosphatidylinositol signaling system, and ErbB signaling following PGB-0-ol treatment. Gene ontology (GO) enrichment analysis demonstrated that these DEGs governed cell cycle, participated in the mitotic spindle and nuclear membrane, and controlled kinase activity at the molecular level. According to the NTA data for GO enrichment, GSEA data for KEGG, drug-gene and disease-gene, PGB-0-ol regulated PI3K/Akt signaling and cell cycle in breast cancer. Overall, our investigation revealed the transcriptomic profile of PGB-0-ol-treated HCC1954 breast cancer cells following PGB-0-ol therapy. Bioinformatics analyses showed that PI3K/Akt signaling and cell cycle was modulated. However, further studies are required to validate the findings of this study.

## 1 Introduction

Breast cancer is the most common type of cancer and the second most common cause of cancer-related deaths in women worldwide (Alkabban and Ferguson, 2022). Breast cancer is classified into three subtypes: the luminal subtypes that express estrogen receptor (ER) and progesterone receptor (PR), the human epidermal receptor 2 (HER2)-positive, and the triple-negative breast cancer (TNBC), which does not express any of the three receptors (Cosar et al., 2022). The discovery of the monoclonal antibody trastuzumab and targeted therapy lapatinib has improved overall survival and disease-free survival in patients with HER2+ breast cancer. However, patients also experience drug resistance leading to relapse. The mechanism of resistance to anti-HER2 therapy is mediated by various mechanisms, including modification of the HER2 binding site so as to create drug impaired binding with HER2, the presence of constitutive activation of HER2 downstream signaling pathways such as the PI3K/AKT and MAPK pathways, and decreased activation of the immune system (de Melo Gagliato et al., 2016, Vernieri et al., 2019); and therefore, the development of newer HER2-targeted drugs needs to continue in order to improve the results of therapy and rates of survival for patients with HER2+ breast cancer (Gleeson et al., 2018).

Curcumin, the main ingredient in turmeric, has been shown to decrease the growth of HER2+ breast cancer cells by blocking downstream signaling, specifically PI3K/AKT pathway (Saxena et al., 2020). Curcumin derivatives have also been shown to decrease HER2+ breast cancer cell proliferation, overcome therapeutic resistance (Lien et al., 2015), and hamper migration (Novitasari et al., 2021). Previously, 2,5-Bis (4-Dihydroxyboryl benzylidene) cyclopentanone—also known as Pentagamaboronon-0 (PGB-0)—was successfully synthesized (Hermawan et al., 2019, Susidarti et al., 2019, Utomo et al., 2017). PGB-0 compounds interact with EGFR and HER2 receptors in molecular docking tests and have exhibited cytotoxic activity against HER2+ breast cancer cells (Utomo et al., 2017); however, poor solubility hinders the development of PGB-0 as a viable drug candidate. PGB-0-ol, which was synthesized later, has better chemical and pharmacological properties than does PGB-0 (Utomo et al., 2022). The cytotoxicity of PGB-0-ol against several breast cancer cells was lower than that of PGB-0, with the strongest cytotoxicity displayed against HCC1954 HER2+ breast cancer cells among the other breast cancer cell subtypes (Utomo et al., 2022). In this study, we used next-generation sequencing technology to explore the molecular mechanism underlying the effect of PGB-0-ol on HCC1954 HER2+ breast cancer cells. Further bioinformatic analyses, such as gene ontology (GO), Kyoto Encyclopedia of Genes and Genomes (KEGG) pathway, disease– and drug–gene association, network topology analysis (NTA), and gene set enrichment analysis (GSEA) were conducted. We believe this study would be useful for the development of PGB-0-ol as an anticancer agent that targets HER2+ breast cancer cells.

## 2 Material and methods

### 2.1 Cell culture

The HCC1954 HER2+ breast cancer cells were obtained from Dr. med. Muhammad Hasan Bashari, M.D., M. PH. (Department of Basic Medicine, Faculty of Medicine, Padjadjaran University, Bandung, Indonesia). HCC1954 cells were grown in RPMI culture medium (Gibco) containing 10 % fetal bovine serum (FBS Qualified, Sigma-Aldrich, USA) and 1 % penicillin–streptomycin (Gibco, Invitrogen, USA) in a 37 °C incubator under 5 % CO_2_. The cells were treated for 72 h with 10 µM the curcumin analog PGB-0-ol, which was synthesized and characterized as previously described (Utomo et al., 2022).

### 2.2 Next generation sequencing

RNA was extracted using RNeasy kits (QIAGEN) according to the manufacturer’s instructions. Using Illumina HiSeq4000 from HiSeq-X sequencing technology, total RNA was prepared for next-generation sequencing. This process included mRNA enrichment, double-stranded cDNA synthesis, end repair, and addition of A overhang and A adaptor, fragment-selection, PCR amplification, testing library quality, and validation. The quality of the cleaned readings was assessed using FastQC version 0.11.9 (https://github.com/s-andrews/FastQC), and the reports were created using MultiQC version 1.1 (https://multiqc.info). Using Kallisto version 0.461, the pseudo-alignment method was used to quantify transcripts using the human genome as a reference (Bray et al., 2016). (GRCh38.p14). a p-value of < 0.05 were set as criteria for the analysis of DEGs in EdgeR version 3.34.0 (Robinson et al., 2010). The fold change (FC) serves as an indicator of whether a gene has undergone upregulation or downregulation.

### 2.3 Gene ontology and Kyoto encyclopedia of genes and genomes pathway enrichment analysis

GO and KEGG pathway enrichment analyses were conducted using ShinyGO 0.76.1 (https://bioinformatics.sdstate.edu/go/) (Ge et al., 2020). DEGs were submitted to ShinyGO as a *Homo sapiens* gene list. Several parameters were set, such as false discovery rate (FDR) < 0.05 was set as the cut-off significant value and pathways to show as 20.

### 2.4 Drug- and disease-gene association analyses

Drug- and disease–gene association analyses were performed using over-representation analysis (ORA) of Webgestalt (https://www.webgestalt.org) (Liao et al., 2019, Wang et al., 2017) using the standard settings of the database. Briefly, DEGs were submitted to WebGestalt and several parameters were selected. For drug–gene association analysis, we used a functional database of drugs and GLAD4U, whereas in disease–gene association analysis, a functional database of diseases and OMIM were selected. FDR < 0.05 was selected as the cut-off significant value.

### 2.5 Network topology analysis (NTA)

WebGestalt was used to perform Network Topology Analysis (NTA) on the PPI BIOGRID functional database (Wang et al., 2017). The top 10 highlighted seed genes and the enriched GO term were selected from the network using a cutoff of 0.05 false discovery rate (FDR).

### 2.6 Gene set enrichment analysis (GSEA)

GSEA was conducted on DEGs using Webgestalt (https://www.webgestalt.org) (Liao et al., 2019, Wang et al., 2017) using the standard settings of the database. Briefly, DEGs and log FC were submitted to WebGestalt, and several parameters were selected such as the organism of interest: *Homo sapiens*; methods of interest: GSEA; and functional database: KEGG pathway and GO. FDR < 0.05 was selected as the cut-off significant value. The normalized enrichment score (NES) was produced by adjusting the enrichment score for each gene set to take the size of the set into account (Subramanian et al., 2005). In this context, the color blue is used to symbolize a positive correlation or a value more than 0, whilst the color orange is employed to depict a negative correlation or a number less than 0. The lowest section of the visual representation illustrates the enrichment plot corresponding to each category (Liao et al., 2019, Wang et al., 2017).

## 3 Results

### 3.1 Next generation sequencing

**Fig. 1:**
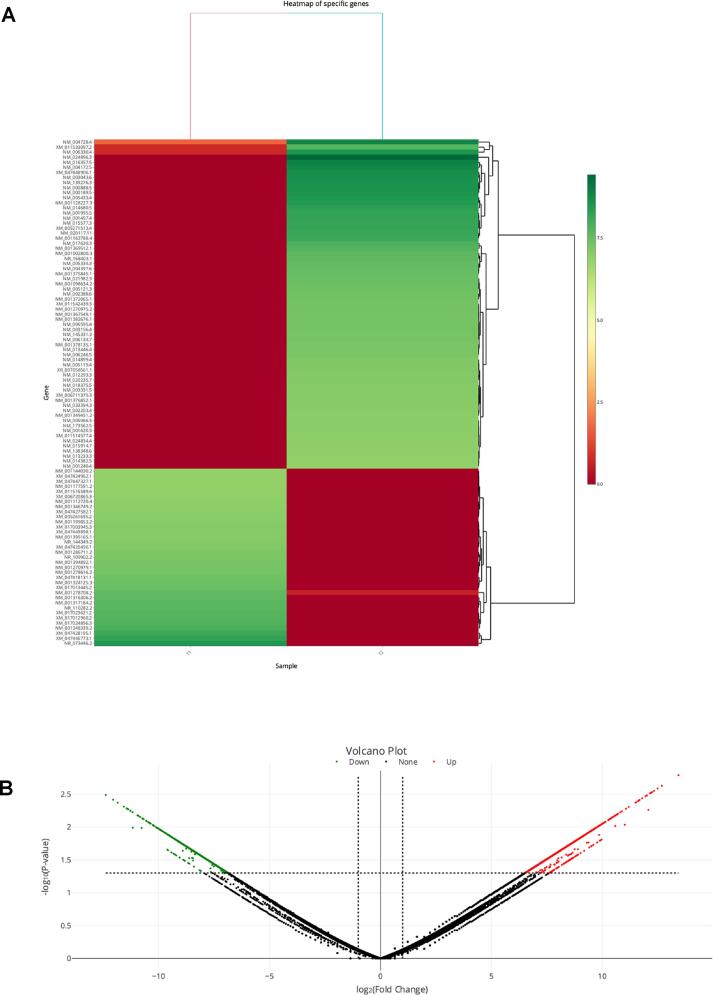
Results of the next-generation sequencing of mRNA of dimethyl sulfoxide (DMSO)-and PGB-0-ol- treated HCC1954 cells. (A) Hierarchical clustering heatmap of the top 100 differentially expressed genes (DEGs). (B) Volcano plot of the DEGs. The log2 FC value is shown on the x-axis, while the y-axis represents the mean expression value of negative log 10 (adjusted p-value). The upregulated and downregulated DEGs are depicted as red and green dots, respectively. (For interpretation of the references to color in this figure legend, the reader is referred to the web version of this article.)

To explore the molecular mechanism underlying HCC1954 HER2+ breast cancer cell inhibition by PGB-0-ol, we first performed next-generation sequencing. A total of 2,263 DEGs consisting of 1,459 upregulated and 804 downregulated genes were detected in the samples (Fig. 1A; Supplementary Table 1). Significant DEGs identified in the PGB-0-ol and DMSO-control groups are shown in the hierarchical heatmap (Fig. 1A, Table 1) and volcano plot (Fig. 1B).

**Table 1:** Top 10 upregulated dan downregulated DEGs.

No	Gene_Symbol	log2 Fold Change (FC)	Gene name	Notes
1	*ERMP1*	13.4263705	Endoplasmic reticulum metallopeptidase 1	Upregulation
2	*LIMA1*	12.6777864	LIM domain and actin-binding protein 1	Upregulation
3	*SLC1A3*	12.4863452	Solute Carrier Family 1 Member 3	Upregulation
4	*ATP13A3*	12.3236714	ATPase 13A3	Upregulation
5	*SLC6A6*	12.2401481	Solute Carrier Family 6A Member 6	Upregulation
6	*STAT3*	12.1922908	Signal transducer and activator of transcription 3	Upregulation
7	*ITGB6*	12.1787374	Integrin beta-6	Upregulation
8	*HK2*	12.1380468	Hexokinase-2	Upregulation
9	*LYPLA1*	12.0691449	Acyl-protein thioesterase 1	Upregulation
10	*YES1*	12.0011934	YES Proto-Oncogene 1	Upregulation
11	*PSMD2*	−11.162358	26S proteasome non-ATPase regulatory subunit 2	Downregulation
12	*ITGA6*	−11.171192	Integrin alpha-6	Downregulation
13	*CDH1*	−11.270941	Cadherin-1	Downregulation
14	*PAFAH1B2*	−11.272435	Platelet-activating factor acetylhydrolase IB subunit beta	Downregulation
15	*EIF3CL*	−11.371921	Eukaryotic translation initiation factor 3 subunit C-like protein	Downregulation
16	*LYPLA1*	−11.386476	Acyl-protein thioesterase 1	Downregulation
17	*NUFIP2*	−11.440617	Nuclear fragile X mental retardation-interacting protein 2	Downregulation
18	*SDCBP*	−11.561602	(Syndecan Binding Protein	Downregulation
19	*XPOT*	−11.845155	Exportin-T	Downregulation
20	*IARS1*	−12.381294	Isoleucine--tRNA ligase	Downregulation

### 3.2 KEGG pathway and GO enrichment analysis

**Fig. 2:**
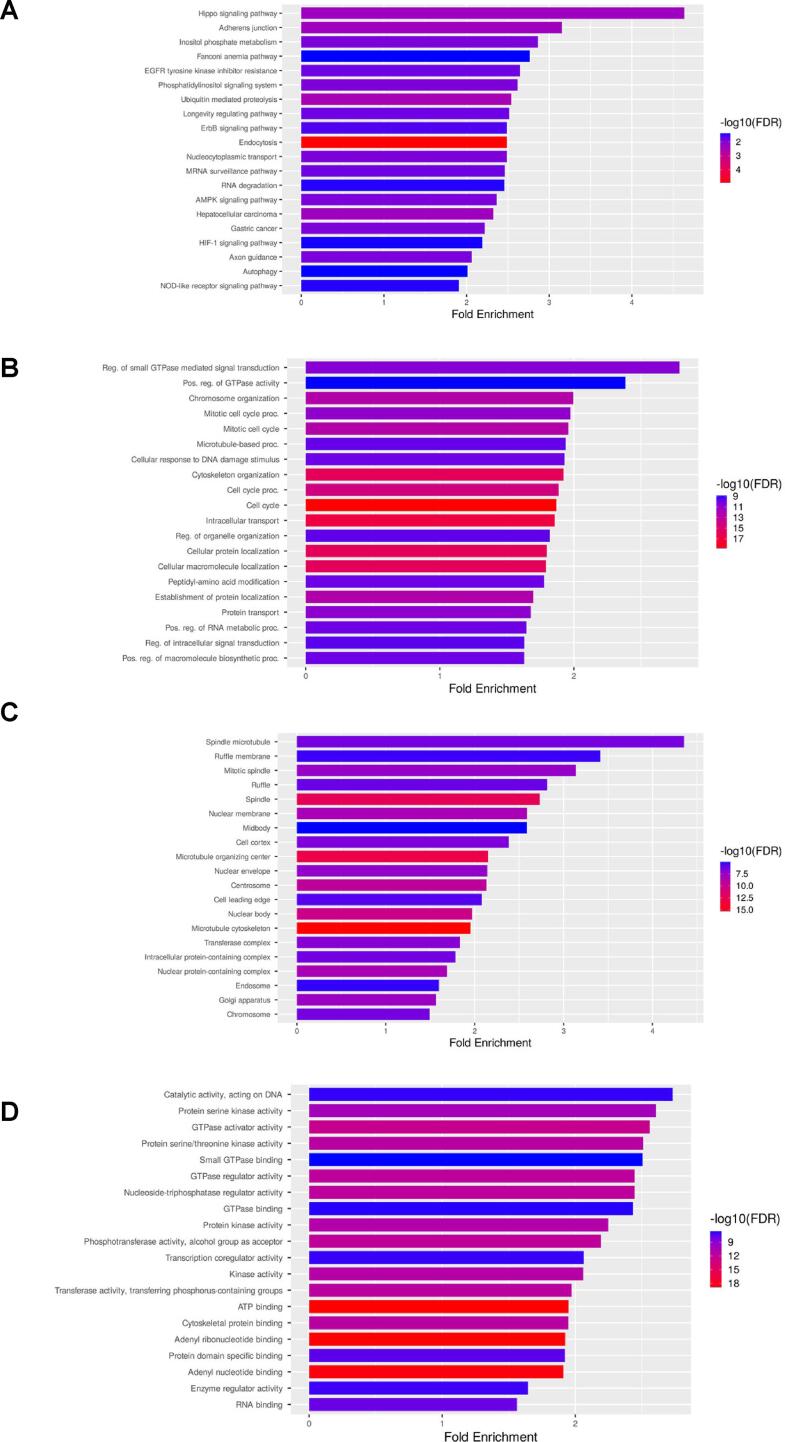
Gene ontology and Kyoto Encyclopedia of Genes and Genomes (KEGG) pathway enrichment analysis of the DEGs between DMSO- and PGB-0-ol- treated HCC1954 breast cancer cells. (A) Biological process (B) Cellular component (C) Molecular function (D) KEGG pathway.

To clarify the probable mechanism underlying the effect exerted by PGB-0-ol on HER2+ breast cancer cells, the DEGs were processed for functional annotation using KEGG pathway and GO enrichment analyses. DEGs were analyzed using the KEGG pathway to explore the DEGs that were activated or suppressed in various classes of pathways. DEGs regulated several pathways, such as Hippo signaling, inositol phosphate metabolism, EGFR tyrosine kinase inhibitor resistance, Phosphatidylinositol signaling system, and ErbB signaling (Fig. 2A). GO enrichment analysis was classified into three categories: biological process, cellular component, and molecular function, and the top 20 results in each category are shown. DEGs regulated biological processes through several mechanisms, such as positive regulation of GTPase activity, mitotic cell cycle, cellular response to DNA damage stimulus, cell cycle, and regulation of intracellular signal transduction (Fig. 2B). DEGs were also located in the cellular components of the spindle microtubule, ruffle membrane, mitotic spindle, and nuclear membrane (Fig. 2C). Moreover, DEGs were involved in the molecular functions of catalytic activity acting on DNA, protein serine/threonine kinase activity, protein kinase activity, and enzyme regulator activity (Fig. 2D).

### 3.3 Drug- and disease–gene association analyses

**Fig. 3:**
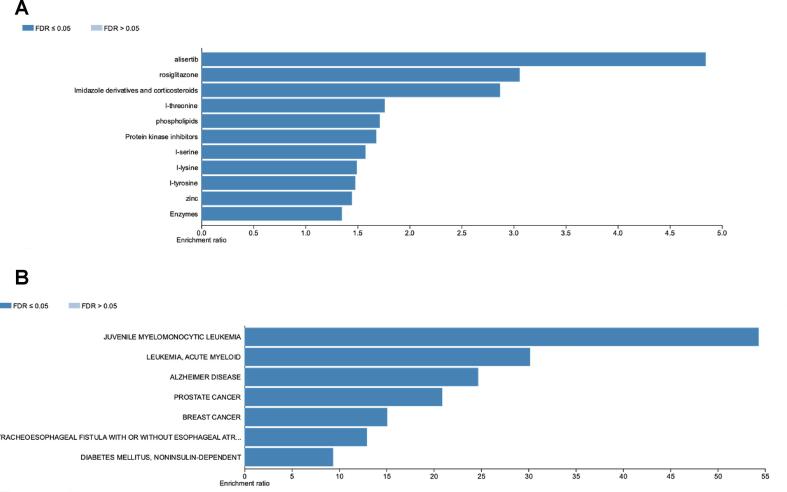
DEG–disease association (A) and DEG–drug association (B) analysis was conducted with over-representation analysis (ORA) of Webgestalt.

The DEGs were further analyzed to determine the association between DEGs, disease, and drugs. The results revealed the association of DEGs with several drugs, such as alisertib, rosiglitazone, phospholipids, and protein kinase inhibitors (Fig. 3A). The DEGs were also associated with several disease such as prostate and breast cancer (Fig. 3B).

### 3.4 Network topology analysis (NTA)

**Fig. 4:**
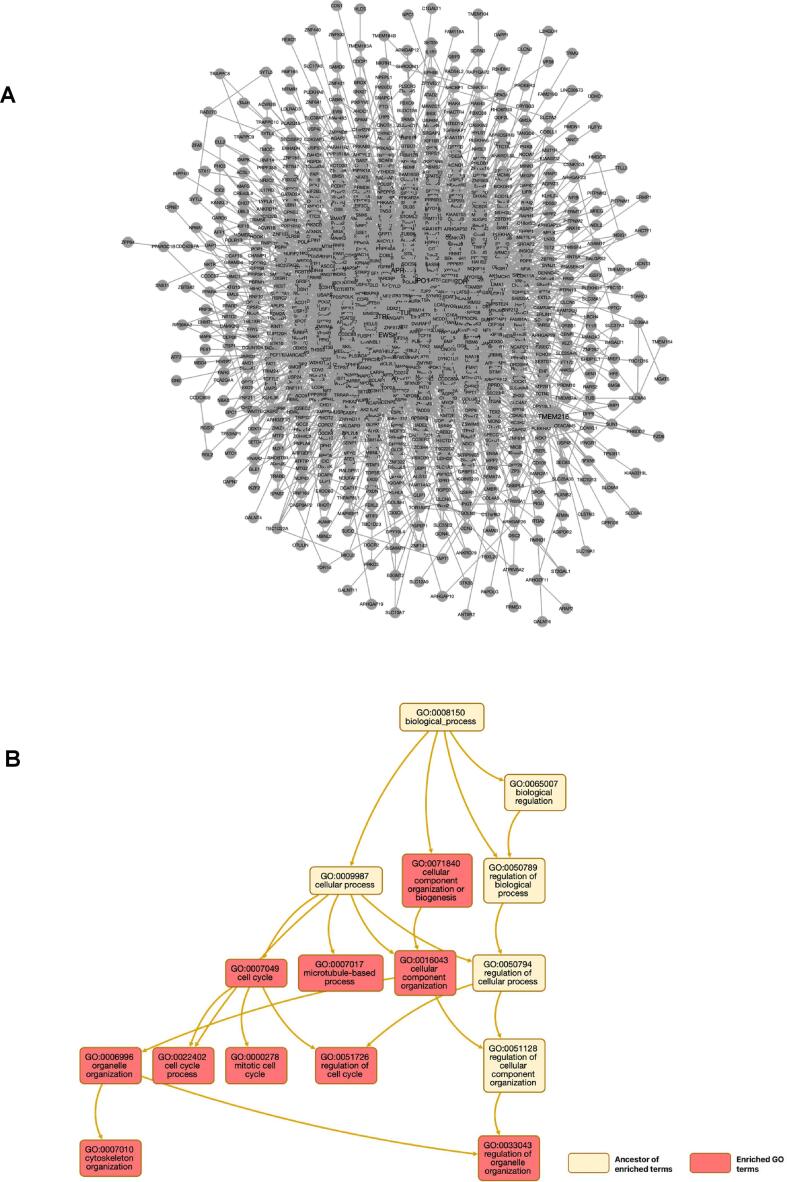
(A). Network Topology Analysis (NTA) and (B) GO enrichment of the NTA, as analyzed using WebGestalt.

NTA is vital for understanding the intricate interactions between genes in biological systems because it allows us to identify key genes that are required for biological systems to function.The protein network from NTA of DEGs resulted in top ten seed genes, including *APP*, *XPO1*, *EGFR*, *CDH1*, *CUL3*, *EFTUD2*, *BRCA1*, *EWSR1*, *TMEM216*, and *BTRC* (Fig. 4A upper part). Additional GO enrichement of NTA revealed that DEGs are linked to several GO like cell cycle, mitotic cell cycle, regulation of cell cycle, cellular component organization, microtubule-based process, cellular component organization or biogenesis, and regulation of organelle organization (Fig. 4B lower part, Supplementary Table 2).

### 3.5 Gene set enrichment analysis (GSEA)

**Fig. 5:**
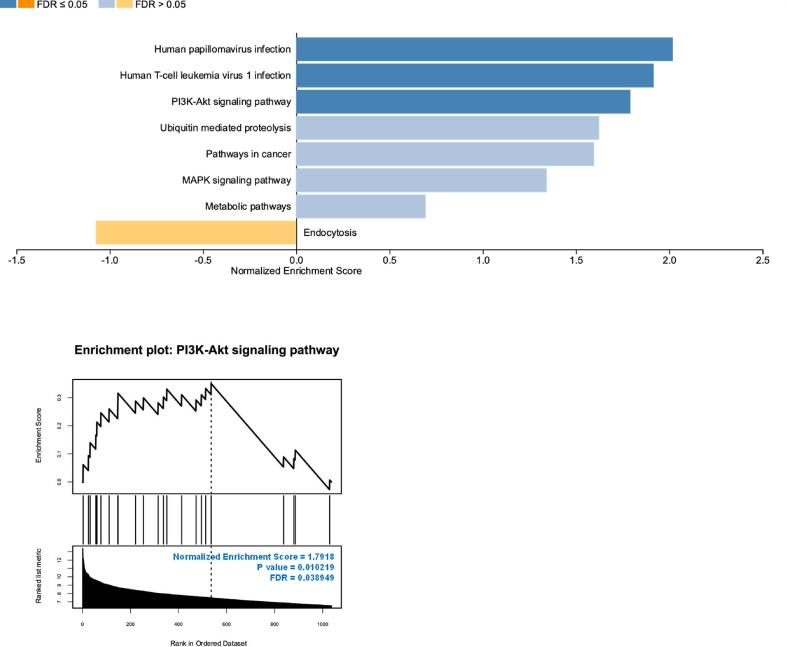
Gene Set Enrichment Analysis (GSEA) results of the DEGs, as analyzed using WebGestalt. Upper part of the figure showed the bar plot of the enrichment. The length of the bar reflects the normalized enrichment score (NES) value, and the color scale depicts the false discovery rate (FDR) value. Specifically, a positive correlation or a value more than 0 is depicted as blue, while a negative correlation or a value less than 0 is represented as orange. The lower portion of the graphic displays the enrichment plot for each category. (For interpretation of the references to color in this figure legend, the reader is referred to the web version of this article.)

On identifying the related pathways and processes, GSEA was used to evaluate the gene expression data from a large-scale experiment. The enriched KEGG pathway by GSEA showed that DEGs were positively enriched for PI3K-Akt signaling pathway with NES = 1.7918 (Fig. 5, Supplementary Table 3). This suggests that PGB-0-ol regulates the PI3K-Akt signaling pathway. We identified 19 genes that were enriched in the PI3K/Akt signaling pathway: *BRCA1, CREB3L4, CSF1, EGFR, ERBB2, ITGA2, ITGB6, JAK2, LAMA3, MET, PDPK1, PIK3CB, PKN2, PPP2R2A, PPP2R5E, PRKAA1, PTK2, RAF1,* and *TSC2* (Table 2).

**Table 2:** DEGs that were enriched as PI3K/Akt pathway based on analysis using GSEA.

No	Gene_Symbol	log2 Fold Change (FC)	Gene name	Notes
1	*BRCA1*	8.37551037	Breast cancer type 1 susceptibility protein	Upregulation
2	*CREB3L4*	7.91565436	Cyclic AMP-responsive element-binding protein 3-like protein 4	Upregulation
3	*CSF1*	8.24781768	Macrophage colony-stimulating factor 1	Upregulation
4	*EGFR*	7.94339935	Epidermal growth factor receptor	Upregulation
5	*ERBB2*	9.19493812	Receptor tyrosine-protein kinase erbB-2	Upregulation
6	*ITGA2*	9.95025302	Integrin alpha-2	Upregulation
7	*ITGB6*	12.1787374	Integrin beta-6	Upregulation
8	*JAK2*	9.52988771	Tyrosine-protein kinase JAK2	Upregulation
9	*LAMA3*	8.40906292	Laminin subunit alpha-3	Upregulation
10	*MET*	7.53293458	MET proto-oncogene, Hepatocyte growth factor receptor	Upregulation
11	*PDPK1*	9.59255857	3-phosphoinositide-dependent protein kinase 1	Upregulation
12	*PIK3CB*	9.32718592	Phosphatidylinositol-4,5-bisphosphate 3-kinase catalytic subunit alpha/beta/delta;	Upregulation
13	*PKN2*	7.57492867	Serine/threonine-protein kinase N2	Upregulation
14	*PPP2R2A*	−10.093901	Serine/threonine-protein phosphatase 2A 55 kDa regulatory subunit B alpha isoform	Downregulation
15	*PPP2R5E*	10.2607402	Serine/threonine-protein phosphatase-2A 56 kDa regulatory subunit epsilon isoform	Upregulation
16	*PRKAA1*	8.01051355	5′-AMP-activated protein kinase catalytic subunit alpha-1	Upregulation
17	*PTK2*	7.67761434	Focal adhesion kinase 1	Upregulation
18	*RAF1*	7.54505814	RAF proto-oncogene serine/threonine-protein kinase	Upregulation
19	*TSC2*	−7.8793706	Tuberin	Downregulation

## 4 Discussion

This study revealed the mechanism underlying the action exerted by PGB-0-ol in HCC1954 cells using transcriptomics followed by bioinformatics analysis. We used three enrichment approaches, ORA (for GO, KEGG pathway, and disease– and drug–gene association analyses), NTA, and GSEA. The utilization of ORA has been employed for the purpose of discovering biological pathways and activities that are disproportionately represented within a given gene list (Wieder et al., 2021), however, it is important to note that ORA does not provide a comprehensive depiction of the relationship between genes and their corresponding products. The GSEA method, as described by (Subramanian et al., 2005), is capable of identifying discrepancies in pathways and functions between two groups, as well as detecting interactions among genes and their corresponding products, however, it does not provide a comprehensive understanding of the overall architecture of the gene network. This limitation is addressed by the NTA approach proposed by Huang et al. (Huang et al., 2018). Since the three approaches followed different algorithms, the findings from each approach are cross-verified.

The results of GO enrichment analysis showed that DEGs regulated cellular processes including cell cycle in organelles such as the mitotic spindle and nuclear membrane and control the molecular function of kinase activity. KEGG enrichment results showed the regulation of the control of phosphatidylinositol signaling system, and ErbB signaling upon PGB-0-ol treatment. According to the NTA data for GO enrichment, GSEA data for KEGG, drug-gene and disease-gene, PGB-0-ol regulated PI3K/Akt signaling and cell cycle in breast cancer.

*BRCA1, CREB3L4, CSF1, EGFR, ERBB2, ITGA2, ITGB6, JAK2, LAMA3, MET, PDPK1, PIK3CB, PKN2, PPP2R2A, PPP2R5E, PRKAA1, PTK2, RAF1,* and *TSC2* were enriched in the PI3K/Akt signaling pathway. *BRCA1* encodes the breast cancer type 1 susceptibility protein, a regulator of DNA repair and the G2/M cell cycle checkpoint, the mutation of which leads to reduced expression, loss of function, and accelerated breast cancer progression (Ma et al., 2020). The PI3K/Akt pathway is activated by changes in gene expression, including reduced *HER2* amplification (Chung et al., 2022). PI3K/Akt signaling regulates BRCA1 phosphorylation in T47D (Ma et al., 2020) cells with low HER2 expression (Mota et al., 2017). However, coincident *BRCA* mutations and HER2 overexpression have been observed (Tomasello et al., 2022). Therefore, the BRCA1-HER2-PI3K/Akt signaling axis and the effects of PGB-0-ol are uncertain. *CREB3L4* encodes the cyclic AMP-responsive element-binding protein 3-like protein 4, a transcription factor responsible for breast cancer progression (Pu et al., 2020). Anti-HER2-targeted therapy has downregulated *CREB3L4* expression in breast cancer samples (Angus et al., 2021). Additionally, CREB4 was observed to moderate glioma proliferation by regulating PI3K/Akt (Li et al., 2021); however, the mechanism of CRB3L4 inhibition in HER2+ breast cancer cells by PGB-0-ol requires further investigation.

*CSF1* encodes macrophage colony-stimulating factor 1, a cytokine that controls immune responses (Sauter et al., 2016) and promotes breast cancer progression (Ding et al., 2016). CSF1 activates its receptor (CSF1R) and subsequently activates PI3K and ERK signaling in HER2+ breast cancer cells (Morandi et al., 2011). The role and mechanism of PGB-0-ol in inhibiting CSF1-PI3K/Akt signaling in HER2+ breast cancer cells need to be explored. *EGFR* encodes the epidermal growth factor receptor, or HER1, which, together with HER2 and other EGFR family members, can activate downstream signaling cascades such as PI3K/Akt and MAPK (Dwivedi et al., 2019).

*ITGA2* encodes integrin alpha-2, a collagen receptor observed on platelets and epithelial cells, and is abundantly expressed in normal epithelial cells (Ding et al., 2015). The loss of ITGA2 is involved in metastasis in colon cancer (Robertson et al., 2009) and breast cancer cells (Ding et al., 2015). Overexpression of *ITGA2* increases the aggressiveness of esophageal squamous cell carcinoma through the Akt signaling pathway (Huang et al., 2021). *ITGB6* encodes integrin beta-6, which regulates epithelial-to-mesenchymal transition (EMT) in wound healing and carcinogenesis (Zheng et al., 2021). High integrin 6 expression, in conjunction with the Rho-Rac pathway, has been associated with poor prognosis in HER2 + breast cancer (Desai et al., 2016). PI3K/Akt signaling activation by transmembrane collagen XVII promotes integrin-dependent migration in invasive squamous cell carcinoma (Löffek et al., 2014). Accordingly, the role of ITGA2/ITGB6-PI3K/Akt in HER2+ breast cancer cells and its modulation by PGB-0-ol warrant further investigation.

*JAK2* encodes the tyrosine-protein kinase JAK2, which controls cell development and differentiation (Gnanasambandan and Sayeski, 2011). Previous studies have emphasized the crosstalk between PI3K, HER2, JAK2, and IL-8 signaling targets for metastatic breast cancer therapies (Britschgi et al., 2013). *LAMA3* encodes laminin subunit alpha-3, which plays a vital role in growth by interacting with other components of the extracellular matrix (Feng et al., 2021) and mediates cell proliferation, migration, and invasion in pancreatic ductal adenocarcinoma cells (Huang and Chen, 2021). LAMB3 has been shown to regulate the PI3K/Akt signaling system and promote apoptotic, proliferative, invasive, and metastatic characteristics in pancreatic cancer (Zhang et al., 2019). *MET* encodes the MET proto-oncogene hepatocyte growth factor receptor, a member of the tyrosine kinase receptor family of proteins that drive cell migration, invasion, and survival via canonical Ras, MAPK, PI3K/Akt, and b-catenin signaling pathways (Rivas et al., 2022). The MET-PI3K/Akt axis is involved in breast cancer resistance to doxorubicin (Kyeong-Ah et al., 2015) and trastuzumab resistance in HER2 overexpressing breast cancer cells (Shattuck et al., 2008). Accordingly, the role of JAK2 and LAMA3 in HER2+ breast cancer cells and their modulation by PGB-0-ol should be investigated further.

*PDPK1* encodes the 3-phosphoinositide-dependent protein kinase 1, which activates Akt in PI3K/Akt signaling (Park et al., 2009). Increased PDPK1 expression promotes gemcitabine resistance in pancreatic adenocarcinoma cells (Li et al., 2018) and radiation resistance in hepatocellular carcinoma (Bamodu et al., 2020). *PIK3CB* encodes phosphatidylinositol-4,5-bisphosphate 3-kinase catalytic subunit beta, or p110β, which is important for the activation of PI3K/Akt signaling (Qu et al., 2021). PIK3CB plays a critical role in the progression of esophageal cancer cells (Pan et al., 2022) and the metastasis of pancreatic cancer cells (Qu et al., 2021). *PKN2* encodes a serine/threonine-protein kinase N2, a member of the protein kinase C subfamily (PKC) (Cheng et al., 2018), and is a regulator of cancer cell cycle progression, migration, and invasion (Lachmann et al., 2011, Schmidt et al., 2007). MTOR signaling activates PKN2 to control PI3KC2b phosphorylation in the PI3K/Akt signaling pathway (Wallroth et al., 2019). *PPP2R2A* encodes the serine/threonine protein phosphatase 2A regulatory subunit B alpha isoform, which is involved in mitosis (Liang et al., 2017). PPP2R2A acts as a tumor suppressor gene, and suppression of PPP2R2A increases the proliferation of leukemia cells (Ruvolo et al., 2014) and human colon cancer cells (Liang et al., 2017). Akt-dependent Pp2a activity is necessary for establishing the epidermal barrier during late embryonic growth (O'Shaughnessy et al., 2009). *PPP2R5E* encodes the serine/threonine protein phosphatase-2A regulatory subunit epsilon isoform involved in the PI3K/Akt, MAPK, and Ras signaling pathways and in the regulation of cancers, including chronic myelogenous leukemia (Perrotti and Neviani, 2013). *PPP2R5B* and *PPP2R5C* are Protein phosphatase 2A (PP2A) regulatory subunits that dephosphorylate Akt (Rocher et al., 2007). PPP2R5E downregulation enhances CRC cell viability, colonosphere formation, and migration (Santos et al., 2023). The roles of PDPK1, PKN2, PPP2R2A, and PPP2R5E in PI3K pathway regulation of HER2+ breast cancer progression and inhibition by PGB-0-ol remain unclear.

*PRKAA1* encodes 5′-AMP-activated protein kinase (AMPK) catalytic subunit alpha-1 and the serine/threonine protein kinase family and functions as a cell energy sensor in eukaryotic cells, regulating intracellular nutrition and energy levels via glucose and lipid metabolic pathways (Gleason et al., 2007). PRKAA1 is a key regulatory kinase in lung cancer-targeted therapies (El-aarag et al., 2017). PRKAA1 increases growth and blocks apoptosis in gastric cancer cells by inducing the JNK1 and Akt pathways (Zhang et al., 2020). Moreover, PRKAA1 enhances cell survival, colony formation, and glycolysis while inhibiting apoptosis by increasing the redox equilibrium in gastric cancer cells (Zhang et al., 2022). *PTK2* encodes protein tyrosine kinase 2, also known as focal adhesion kinase (FAK), a non-receptor tyrosine kinase that plays an important role in integrin-mediated signaling (Zhang et al., 2023). Oxidative stress-induced FAK activation promotes the progression of uterine serous carcinoma (Lopez-Mejia et al., 2023). FAK and Akt interact to maintain stem cell properties and migration in human colorectal cancer cells, and downregulation of FAK leads to decreased phosphorylation of Akt and subsequent downregulation of cancer stem cell markers and spheroid formation (Xu et al., 2023). *RAF1* encodes the RAF proto-oncogene serine/threonine-protein kinase, a protein that belongs to the RAS/RAF/MEK/ERK signaling system, which controls cell migration, apoptosis, and differentiation (Tian et al., 2018). The Raf/MEK/ERK and PI3K/PTEN/Akt/mTOR pathways interact to regulate cancer cell growth and sensitivity to therapies (Steelman et al., 2011). *TSC2* encodes the growth-inhibitory protein tuberin that regulates protein synthesis and cell cycle progression (Fidalgo da Silva et al., 2023). Phosphorylation of tuberin via PI3K/Akt signaling leads to its inactivation and enhances the progression of human endometrial adenocarcinoma cells (Sales et al., 2004). The Akt/tuberin/mTOR pathway regulates DNA damage and repair mechanisms, potentially exposing diabetic kidneys to RCC development of renal cell carcinoma (Habib and Liang, 2014). In the Mexican population, a new mutation in the TSC2 gene protects against colorectal cancer (González-Villaseñor et al., 2022). The functions of PRKAA1, PTK2, and TSC2 in PI3K/signaling governing HER2+ breast cancer development and suppression by PGB-0-ol remain unknown and require further investigation.

The upregulation of HER2 in breast cancer cells results in the initiation of its subsequent signaling pathways, including PI3K/Akt (Ruiz-Saenz et al., 2018) and RAS/RAF/MAPK (Goltsov et al., 2014), which in turn promote cellular proliferation, survival, angiogenesis, and metastasis (Wilks, 2015). PI3K/Akt signaling pathway is one of the most common overactivated pathway in breast cancer (Ellis and Ma, 2019). Activation of PI3K/Akt signalling leads to modulation of regulator of cell cycle, cyclins, cyclin-dependent kinases, and cyclin-dependent kinase inhibitors (Chang et al., 2003). In addition, activation of PI3K/Akt signaling leads to breast cancer cells resistance to anti HER2 theraphy such as trastuzumab, lapatinib, afaitinib, and pelitinib (Dong et al., 2021). A previous study showed that combination treatment in PIK3CA-mutant HER2+ breast cancer cells using PI3K/Akt inhibitors and and anti HER2 lapatinib, increase sensitivity of lapatinib (Fujimoto et al., 2020). Results of this study is supported by a previous study that showed induction of S-phase cell cycle arrest by the PGV-0 in HCC1954 cells (Angraini et al., 2019). A previous study showed that curcumin inhibited cell growth in BT474 HER2+ breast cancer cells by downregulating PI3K/Akt signaling in vitro and xenograft (Lai et al., 2012). In addition, curcumin analogue, namely 1,5-bis(4-hydroxy-3-((4-methylpiperazin-1-yl)methyl)phenyl)penta-1,4-dien-3-one induced apoptosis and cell cycle arrest in MCF-7 and MDA-MB 231 human breast cancer cells by targeting the PI3K/Akt signaling (Badr et al., 2018). A recent study showed that demethoxycurcumin induces apoptosis in HER2 overexpressing bladder cancer cells through degradation of HER2 and inhibiting the PI3K/Akt pathway (Kao et al., 2021).

The ORA of disease–gene association showed that DEGs were enriched for breast cancer, whereas drug–gene association analysis revealed the correlation of DEGs with protein kinase inhibitors and alisertib. Alisertib is a serine/threonine protein kinase Aurora A kinase (AURKA) inhibitor that disrupt the chromosome aggregation therefore inhibits cell cycle in G2/M phase and cell proliferation, and induces apoptosis via inhibition of p38/MAPK and Akt signaling pathways (Li et al., 2015). Activation of AURKA by phosphorylation leads to activation of its downstream such as PI3K/Akt, NFkB, p53/MDM2-cell cycle progression (Durlacher et al., 2016). A clinical trial of alisertib in patients with breast cancer has been conducted and the results revealed only 18 % of patients involved in the clinical trial showed partial response (Melichar et al., 2015).

The major limitation of this study was the small sample size. Another limitation was the use of only one cell line for the experiment. A small sample size might mean that the results cannot be generalized or have a chance for false positives; therefore, further investigations are warranted to confirm the findings of this study. Overall, this study revealed the transcriptomic profile of PGB-0-ol-treated HCC1954 breast cancer cells. Bioinformatic analyses revealed modulation of PI3K/Akt signaling and cell cycle was modulated upon treatment with PGB-0-ol. The present study has elucidated the transcriptomics profile of HCC1949 breast cancer cells following therapy with PGB-0-ol. Nevertheless, in cellular processes, there exists a discrepancy between RNA expression and protein expression, indicating a lack of direct proportionality. Consequently, it will become imperative to employ techniques such as 2D electrophoresis or LC-MS/MS-based proteomics in order to investigate protein expression. By doing so, it will be possible to gain insights into the intricate interactions between genes and proteins. Accordingly, further studies are required to validate the molecular mechanisms underlying these effects.

## 5 Conclusion

The results of this study revealed the transcriptomic profile of the PGB-0-ol-treated HCC1954 breast cancer cells. Enrichment analyses showed the modulation PI3K/Akt in cell cycle, which is proposed as the main mechanism through which PGB-0-ol exerts its effect on HCC1954 breast cancer cells. Further investigations are warranted to confirm the findings of this study.

## Availability of data and materials

RNA sequencing data are available in the public database of Gene Expression Omnibus; accession number: GSE218021.

## Funding

This study was supported by the 10.13039/501100002920Research Grant of the Ministry of Education, Culture, Research, and Technology, Republic of Indonesia,through Research Project Number 1619/UN1/DITLIT/DitLit/PT.01.03/2022.

## Authors’ contributions

AH, MK, EM designed the study. AH wrote the manuscript. AH, FW, RYU collected the data. AH analyzed the data. EM and MK supervised the study. All authors read and approved the final manuscript.

## Declaration of Competing Interest

The authors declare that they have no known competing financial interests or personal relationships that could have appeared to influence the work reported in this paper.
